# Refractory obstructive jaundice in a child affected with thalassodrepanocytosis: a new endoscopic approach

**DOI:** 10.1186/1471-230X-10-117

**Published:** 2010-10-13

**Authors:** Gabriele Curcio, Marco Sciveres, Marta Di Pisa, Ilaria Tarantino, Luca Barresi, Silvia Riva, Mario Traina

**Affiliations:** 1 Department of Gastroenterology, IsMeTT, Palermo, Italy; 2 Department of Pediatric Hepatology and Pediatric Liver Transplantation, IsMeTT, Palermo, Italy

## Abstract

**Background:**

Liver involvement, including elevated direct-reacting bilirubin levels, is common in patients with sickle cell disease. Fifty to seventy percent of sickle cell patients have pigmented gallstones due to precipitation of unconjugated bilirubin, and cholelithiasis or choledocholithiasis are common complications. The highest prevalence of these complications occurs in patients with Gilbert's syndrome because of the combined effect of increased bilirubin production and reduced bilirubin-diphosphate-glucuronosyltransferase enzyme activity. Cholelithiasis is also a common complication in patients with thalassemia. Endoscopic removal of choledochal stones does not always resolve the clinical picture, as in cases of dysfunction of the Vater's papilla, increased bile density due to persistently impaired bile flow or distortion of the choledocus due to dilatation, or inflammation secondary to gallstone.

**Case presentation:**

We report here a case of severe and persistent obstructive jaundice in a child affected with thalassodrepanocytosis and Gilbert's syndrome, previously, and unsuccessfully, treated with endoscopic removal of choledochal stones. Deep and thorough biliary washing, and stenting with a new removable polytetrafluoroethylene (PTFE)-covered flared-type stent led to complete resolution of the obstructive jaundice.

**Conclusions:**

This report shows that an aggressive endoscopic approach in this select category of patients can help resolve the severe complication of hemolytic anemia, thus avoiding surgery.

## Background

Liver involvement is a common finding in patients with sickle cell disease. Three principal clinical pictures have been recognized: acute intrahepatic cholestasis, hepatic crisis, and lithiasis [1-4]. Cholelithiasis and choledocholithiasis are common complications in patients with sickle cell disease or thalassemia. The highest prevalence of these complications occurs in patients with Gilbert's syndrome. Endoscopic removal of choledochal stones does not always resolve the clinical picture, as in cases of dysfunction of the Vater's papilla, increased bile density due to persistently impaired bile flow or to distortion of the choledocus due to dilatation, or to inflammation secondary to gallstones.

We report here a case of severe and persistent obstructive jaundice in a child affected with thalassodrepanocytosis and Gilbert's syndrome, previously and unsuccessfully treated with endoscopic removal of choledochal stones. Deep and thorough biliary washing, and stenting with a new removable polytetrafluoroethylene (PTFE)-covered stent led to complete resolution of the obstructive jaundice.

## Case Presentation

A 13-year-old boy, affected with thalassodrepanocytosis (genotype S/β0) and Gilbert's syndrome, presented with cholestatic jaundice. He had no history of obstructive jaundice, and had undergone prophylactic laparoscopic cholecystectomy four years earlier. Liver chemistry tests showed: total/direct reacting bilirubin 69/41 mg/dl, AST 150 U/L (normal: 5-40 U/L), ALT 100 U/L (normal: 10-65 U/L), alkaline phosphatase 165 U/L (normal: 40-134 U/L), and gamma-GT 101 mg/l (normal: 5-85 U/L). A previous ERCP showed choledochal dilation, with an angle in the medium tract, and multiple small intraluminal filling defects, attributable to gallstones. Despite sphincterotomy, gallstone removal and nose-biliary drainage placement, the jaundice worsened and he was referred to our institute.

On admission, the patient was markedly jaundiced, but asymptomatic. He had no history of alcohol intake, viral infections or illicit drug use. His physical examination was remarkable for firm hepatomegaly, and scleral and cutaneous jaundice.

**Figure 1:**
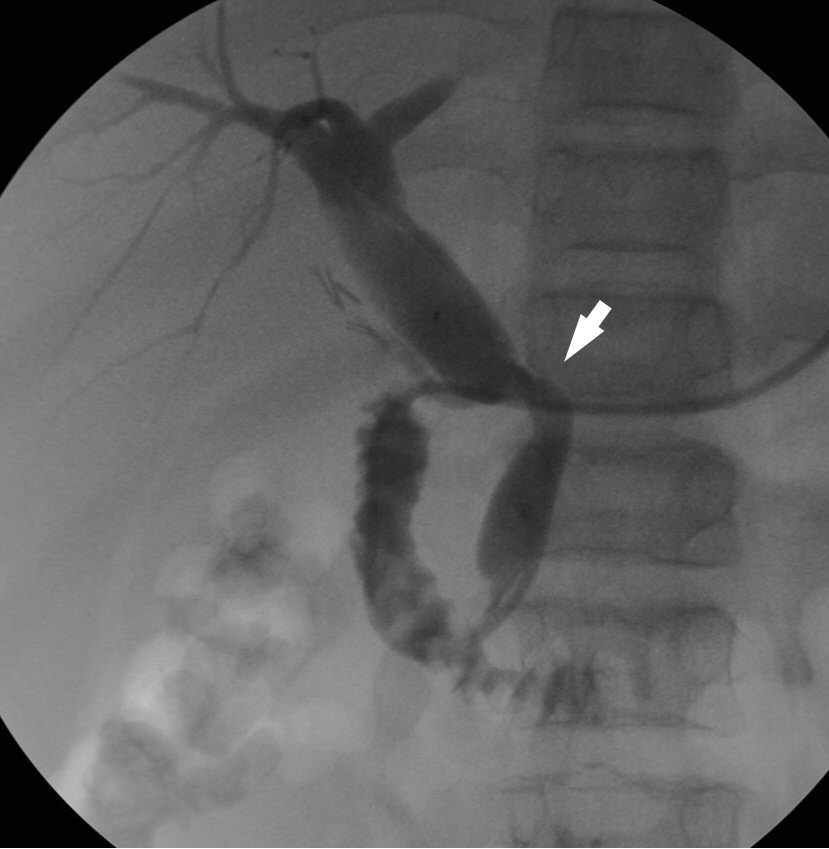
**Dilation of the common bile duct, with an angle in the medial tract (arrow)**.

A cholangiogram through the nose-biliary drainage showed a dilated choledocus, with an angle in the medium tract (Figure 1). Drainage was removed, and abundant dense sludge was removed with a Fogarty balloon catheter. Given the extreme density of the bile, the biliary tree was thoroughly washed with a large amount of sterile physiological solution (approximately 700 cc). In order to achieve a better biliary flow, we decided to place a biliary stent. Given the density of the bile, the dilation and the angle of the choledocus, we decided to place a self-expandable metal stent (SEMS), namely a nitinol polytetrafluoroethylene (PTFE) full-covered flared-type stent, 6 cm in length with 10 mm of diameter, (Niti-S Biliary Covered Stent - Taewoong Medical Co., Korea), and not a plastic one. Compared to plastic stents, metal stents offer the advantage of greater diameter, thus avoiding the risk of migration and stent occlusion, and the additional advantage of radial force, which should correct the angle of the choledocus.

**Figure 2:**
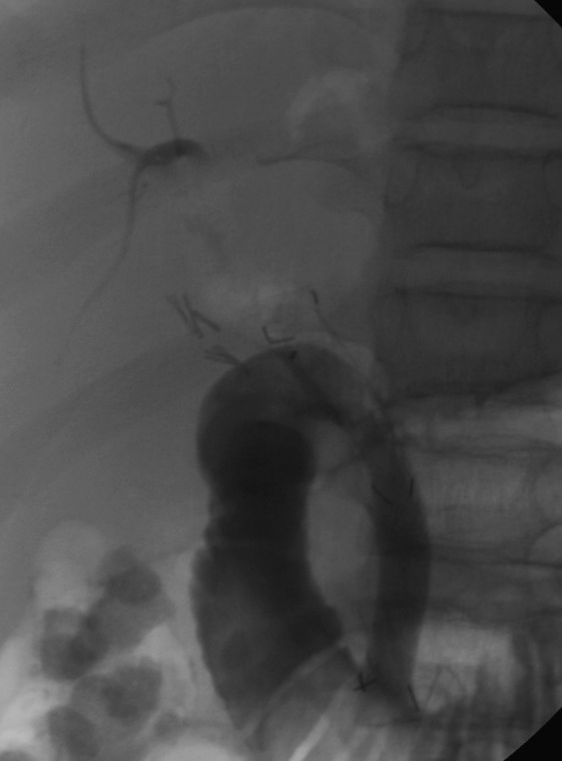
**A PTFE-full covered self-expandable metallic stent is placed in the common bile duct**.

**Figure 3:**
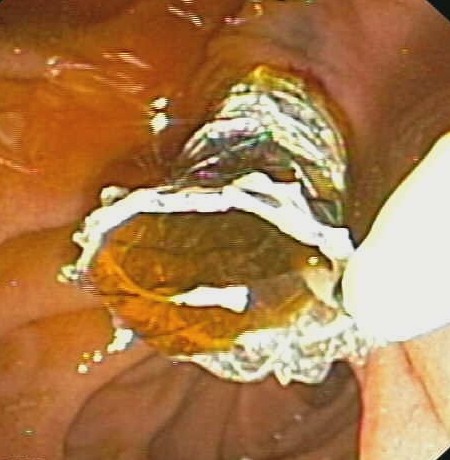
**Metallic stent draining bile into the duodenum**.

The stent was placed in the choledocus, across the angle (Figure 2), draining a large amount of dense bile (Figure 3). The procedure was uncomplicated.

Over the following days, a rapid decrease of bilirubin was observed, and the patient was discharged home in good general conditions, with laboratory tests showing bilirubin tot/dir 9.29/0.37 mg/dl (0-1.5 mg/dL), AST/ALT 82/68 U/L (normal: 5-40/65 U/L), alkaline phosphates 234 U/L (40-134 U/L), and gamma-GT 19 mg/l (5-85 U/L).

**Figure 4:**
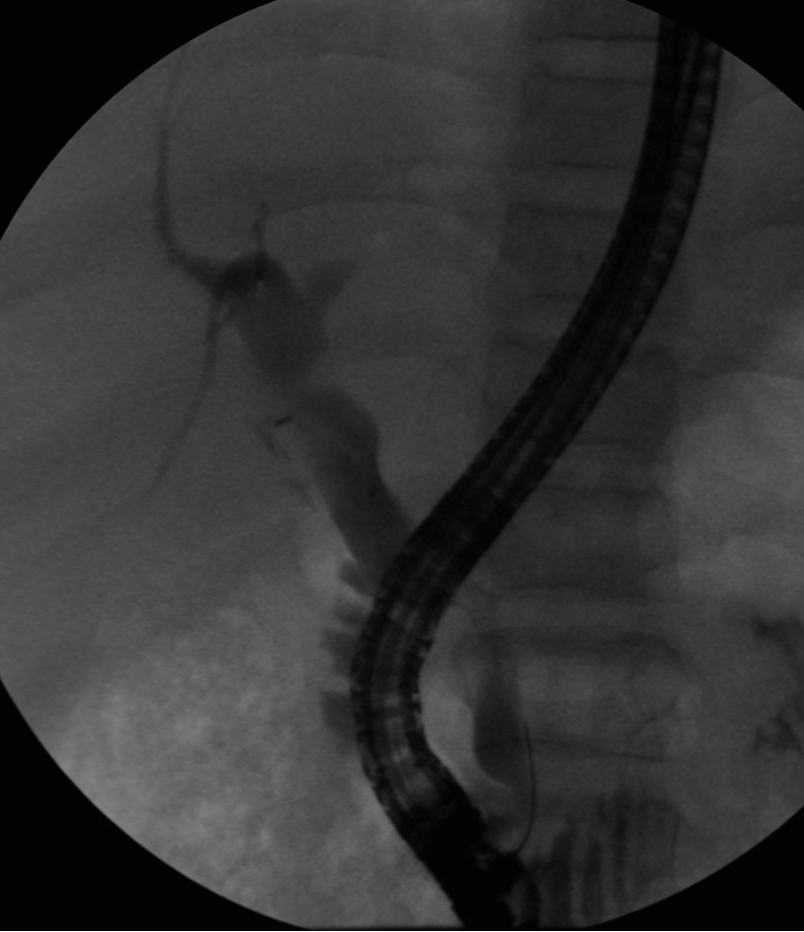
**Cholangiography after stent removal, showing resolutiom of biliary angle, no filling defects, and good biliary drainage**.

The biliary stent was extracted 2 months later, and a cholangiogram showed no filling defects, and resolution of the biliary angle (Figure 4). The patient had no further episodes of cholestatic jaundice during the one year of follow-up. The indirect-reacting bilirubin level remained high, fluctuating between 7 and 15 mg/dl, with normal direct-reacting fraction, and no evidence of biliary stone recurrence at ultrasonography.

## Discussion

Liver involvement is a common finding in patients with sickle cell disease. Three principal clinical pictures have been recognized: acute intrahepatic cholestasis, hepatic crisis and lithiasis [1-4]. Possible causes of acute intrahepatic cholestasis and hepatic crisis are acute intrasinusoidal sickling, with subsequent obstruction, and hepatic ischemia. Moreover, 50-70% of sickle cell patients have pigmented gallstones due to precipitation of unconjugated bilirubin, and cholelithiasis or choledocholithiasis are common complications [1,5,6]. The highest prevalence of these complications occurs in patients with Gilbert's syndrome because of the combined effect of increased bilirubin production and reduced bilirubin-diphosphate-glucuronosyltransferase (UGT1-A1) enzyme activity [7,8]. Cholelithiasis is also a common complication in patients with thalassemia. To avoid these clinical consequences, elective cholecystectomy with appropriate preoperative preparation is recommended for patients in whom gallstones develop [9,10].

Cholelithiasis and choledocholithiasis can cause symptomatic obstructive jaundice, with abdominal pain that mimics painful vaso-occlusive events. A correct diagnosis is critical, though challenging [11]. Endoscopic removal of choledochal stones does not always resolve the clinical picture, as in cases of dysfunction of the Vater's papilla, increased bile density due to persistently impaired bile flow or to distortion of the choledocus due to dilatation, or inflammation secondary to gallstones.

In this case, severe and persistent obstructive jaundice in a child affected with thalassodrepanocytosis and Gilbert's syndrome was successfully resolved with deep and thorough biliary washing, and stenting with a new removable polytetrafluoroethylene (PTFE)-covered stent.

## Conclusions

This report shows that obstructive jaundice in patients with chronic biliary lithiasis can persist after gallstone removal because of bile density and deformation of chronically dilated choledocus.

An aggressive endoscopic approach, with thorough biliary cleansing together with PTFE full-covered removable SEMS stenting in this select category of patients can help resolve the severe complication of hemolytic anemia, thus avoiding surgery.

## Competing interests

The authors declare that they have no competing interests.

## Authors' contributions

GC and MT were the lead investigators, performed the endoscopy, clinically managed the patient, designed and interpreted the manuscript, reviewed the manuscript, and gave the final approval of the version to be published.

MS, MDP, IT, LB and SR were involved in drafting the manuscript, and critically revising it.

## Consent

Written informed consent was obtained from the parents of the patient for publication of this case report and any accompanying images. A copy of the written consent is available for review by the editor in chief of this journal

## Pre-publication history

The pre-publication history for this paper can be accessed here:

http://www.biomedcentral.com/1471-230X/10/117/prepub
